# Diagnostic accuracy and inter-reader reliability of the MRI Liver Imaging Reporting and Data System (version 2018) risk stratification and management system

**DOI:** 10.4102/sajr.v26i1.2386

**Published:** 2022-05-19

**Authors:** Ranjit Singh, Mitchell P. Wilson, Florin Manolea, Bilal Ahmed, Christopher Fung, Darryn Receveur, Gavin Low

**Affiliations:** 1 Department of Radiology and Diagnostic Imaging, University of Alberta, Edmonton, Canada

**Keywords:** liver, cirrhosis, hepatocellular carcinoma, magnetic resonance imaging, reliability, neoplasm

## Abstract

**Background:**

Hepatocellular carcinoma (HCC) can be diagnosed non-invasively, provided certain imaging criteria are met. However, the recent Liver Imaging Reporting and Data System (LI-RADS) version 2018 has not been widely validated.

**Objectives:**

This study aimed to evaluate the diagnostic accuracy and reader reliability of the LI-RADS version 2018 lexicon amongst fellowship trained radiologists compared with an expert consensus reference standard.

**Method:**

This retrospective study was conducted between 2018 and 2020. A total of 50 contrast enhanced liver magnetic resonance imaging (MRI) studies evaluating focal liver observations in patients with cirrhosis, hepatitis B virus (HBV) or prior HCC were acquired. The standard of reference was a consensus review by three fellowship-trained radiologists. Diagnostic accuracy including sensitivity, specificity, positive predictive value (PPV), negative predictive values (NPV) and area under the curve (AUC) values were calculated per LI-RADS category for each reader. Kappa statistics were used to measure reader agreement.

**Results:**

Readers demonstrated excellent specificities (88% – 100%) and NPVs (85% – 100%) across all LI-RADS categories. Sensitivities were variable, ranging from 67% to 83% for LI-RADS 1, 29% to 43% for LI-RADS 2, 100% for LI-RADS 3, 70% to 80% for LI-RADS 4 and 80% to 84% for LI-RADS 5. Readers showed excellent accuracy for differentiating benign and malignant liver lesions with AUC values > 0.90. Overall inter-reader agreement was ‘good’ (kappa = 0.76, *p* < 0.001). Pairwise inter-reader agreement was ‘very good’ (kappa ≥ 0.90, *p* < 0.001).

**Conclusion:**

The LI-RADS version 2018 demonstrates excellent specificity, NPV and AUC values for risk stratification of liver observations by radiologists. Liver Imaging Reporting and Data System can reliably differentiate benign from malignant lesions when used in conjunction with corresponding LI-RADS management recommendations.

## Introduction

Hepatocellular carcinoma (HCC) is the sixth most common malignancy and the second most common cause of malignancy-related mortality worldwide.^1^ Unlike most other cancers, HCC can be confidently diagnosed non-invasively on imaging without mandatory pathology confirmation provided strict imaging criteria are met.^2^

The Liver Imaging Reporting and Data System (LI-RADS), first released in 2011 by the American College of Radiology (ACR), is an imaging reporting algorithm designed to standardise radiology reporting of HCC in high-risk patients in terms of screening, surveillance, diagnosis and treatment response assessment.^1,3^ The LI-RADS categories have the ability to accurately stratify the probability of HCC and overall malignancy without potential risks of biopsy, including inadequate sampling, haemorrhage and biopsy tract seeding.^4,5^ Accordingly, there has been increasing reliance upon imaging and radiologists for both early and accurate diagnosis of HCC, using a universal reporting language.^6^

However, some literature has questioned the accuracy and reliability of the LI-RADS risk stratification.^6,7^ Furthermore, the updated LI-RADS version 2018 has not been widely validated.^8,9,10,11^ This retrospective study aims to evaluate the diagnostic accuracy and inter-reader reliability of the current LI-RADS version 2018 lexicon amongst board certified fellowship trained body imaging radiologists as compared with an expert consensus reference standard.

## Research methods and design

### Patient selection

The University of Alberta Hospital Picture Archiving and Communication System (PACS) was reviewed for all cases with contrast-enhanced MRI studies evaluating the liver, performed between 01 January 2018 and 31 March 2020. Cases with observations found in patients with a high-risk feature for HCC including cirrhosis, chronic hepatitis B viral infection or current or prior HCC and at least 1 year of cross-sectional imaging follow-up were selected for inclusion. Observations in patients under the age of 18 years, those with absence of high-risk factors and those with cirrhosis caused by non-hepatitis aetiologies were excluded as per the ACR CT/MRI LI-RADS v2018 core guidelines.^7^ For the purposes of this study, only LI-RADS categories 1 to 5 were considered, with cases involving other malignancy (LR-M), tumour in vein (LR-TIV) and treatment response (LR-TR) categories also excluded. The 50 cases included in the study consisted of those lesions with typical representative features for each of the LI-RADS categories. All MRI studies were of good technical quality and in line with the ACR recommendations.^7^

A total of 50 non-consecutive cases were selected by consensus from a Steering Committee of two authors with 6- and 13-years experience. Cases were chosen to represent a mix of classic imaging features and equivocal and challenging features in order to reflect a range of cases, which may be seen in a routine tertiary hospital setting. Only a single lesion per case was considered. When multiple lesions were present on a single case, only the lesion with the highest suspicion score was considered and annotated for review.

### Liver MRI protocol

All liver MRI examinations included in this study were performed by using 1.5-T MRI scanners (GE Healthcare, Milwaukee, Wis; HD, GE Healthcare). Pre-contrast sequences included axial DWI (b values: 0, 50, 150 and 500) with ADC images, axial T2-weighted images with single-shot fast spin echo (FSE) technique, gradient echo (GRE) T1-weighted out-phase and in-phase axial images and axial pre-contrast breath hold fat saturated spoiled-GRE images. Fat saturated post-contrast dynamic images were acquired in late arterial (30–40 s), portal (60–90 s), late portal (120–150 s) and delayed phases (180–210 s and at 300+ s) with breath-hold spoiled-GRE 3D technique in the axial and coronal planes. Where necessary, subtracted images were obtained from the dynamic sequences in order to aid lesion interpretation. Gadobutrol (Gadovist; Bayer Healthcare Pharmaceuticals, Whippany, NJ, United States) was the contrast agent used in all cases. A weight-based dose bolus of gadolinium contrast (0.1 mmol/kg body weight) was injected intravenously, followed by a normal saline flush (20 mL). Contrast material injection was given via a peripheral vein at 5 cc/s.

### Image processing

All cases were randomised using the Microsoft Excel randomisation function and identifiers were removed from each MRI examination. The cases were subsequently networked to the PACS workstation (IMPAX 6 AGFA Healthcare) under an allocated post-randomisation case number. When one or more comparison studies were available, the most relevant comparison was selected and was similarly de-identified and stored on the workstation under the same case identifier.

### Liver lesion imaging atlas

Single images that best depicted each observation (*n* = 50) were captured and stored in their respective case folders on the intuitional PACS (IMPAX 6 AGFA Healthcare) in order to guide the readers and to allow for a targeted assessment of individual observations. The image that most clearly showed each observation was chosen, regardless of the MRI sequence type or post-contrast phase. Each de-identified case folder contained the representative image that depicted the targeted observation including the size of the observation to be used, the MRI sequences pertaining to the targeted observation and comparison studies, where available. No patient identifiers were included in the case folders.

### Image review

All 50 lesions were reviewed and assigned a LI-RADS category by consensus reading of three fellowship-trained body imaging radiologists (G.L., M.P.W., F.M.). Expert consensus for any given score was considered if all three radiologists agreed on the same score. Any disagreement was resolved by consensus re-review of the imaging and discussion. The test cases were then subsequently independently reviewed by three separate fellowship trained readers (B.A., C.F., D.R.), all with five years or more post-fellowship experience in body MR imaging. Prior to testing, each reader was provided with an instruction manual and the official LI-RADS atlas and glossary published by the ACR to be used at any point as a reference tool.^7^ Readers were blinded to the patient history, initial radiology report, consensus LI-RADS score and interpretation of other readers. All 50 cases were independently reviewed, and data were entered into a standardised online data entry web form. For each LI-RADS score, the reader also indicated whether LI-RADS ancillary features for HCC were used to assign that score. The reviewers’ consensus for definitively benign or probably benign liver lesions (LI-RADS 1 or 2) was based on: (1) typical or near typical imaging features for a benign aetiology on MRI and (2) interval size stability of at least 12 months.

### Statistical analysis

Categorical variables were expressed as values and percentages. Continuous variables were expressed as the mean ± standard deviation. Statistical tests included:

- One-way analysis of variance (ANOVA) to evaluate for significant differences in lesion size between the LI-RADS categories
- Diagnostic accuracy measurements including sensitivity, specificity, positive predictive value (PPV) and negative predictive value (NPV) were calculated per LI-RADS category for each individual reader
- Receiver operating characteristic (ROC) analysis was used to evaluate the area under the receiver operating curve (AUC) for each reader
- Fleiss kappa (overall agreement) and weighted quadratic kappa (pairwise agreement) was used to calculate the inter-reader agreement. The kappa (κ) value interpretation as suggested by Cohen was used: κ < 0.20 (poor agreement), κ = 0.21–0.40 (fair agreement), 0.41–0.60 (moderate agreement), 0.61–0.80 (good agreement) and 0.81–1.00 (very good agreement).^8^

All statistical analyses were conducted using IBM Statistical Package for Social Sciences (SPSS) (version 26) and MedCalc (version 19.6.1). A *p*-value of < 0.05 was considered as statistically significant.

### Ethical considerations

This single centre retrospective study was approved by the University of Alberta Health Research Ethics Board (Pro00098131). Patient consent for individual cases was waived as all studies were retrospectively collected from the institutional Picture Archiving and Communication System (PACS) and studies were anonymised prior to review by individual readers.

## Results

There were 50 cases in the study. The mean patient age was 59.9 ± 11.3 years, ranging from 21 to 78 years. There were 38 males (76%) and 12 females (24%). The aetiology of cirrhosis was hepatitis C virus (HCV) in 14 (28%), non-alcoholic steatohepatitis (NASH) in 9 (18%), alcohol in 7 (14%), hepatitis B virus (HBV) in 6 (12%), primary sclerosing cholangitis (PSC) in 4 (8%), autoimmune in 4 (8%) and cryptogenic in 1 (2%). The aetiology of cirrhosis was unknown in the remaining 5 cases (12%). According to the reference standard, there were 6 cases (12%) of LI-RADS 1, 7 cases (14%) of LI-RADS 2, 2 cases (4%) of LI-RADS 3, 10 cases (20%) of LI-RADS 4 and 25 cases (50%) of LI-RADS 5. Aetiologies of the six LI-RADS 1 cases included benign cysts (*n* = 4), haemangioma (*n* = 1) and focal nodular hyperplasia (*n* = 1) and aetiologies of the seven LI-RADS 2 cases included regenerative nodules (*n* = 5), atypical haemangioma (*n* = 1) and transient hepatic intensity difference (THID) (*n* = 1). There were 32 (64%) lesions in the right hepatic lobe and 18 (36%) in the left lobe.

The overall lesion size was 21 mm ± 12 mm with a range from 8 mm to 57 mm. Mean lesion size by LI-RADS category was: 16 mm ± 3 mm for LI-RADS 1, 12 mm ± 3 mm for LI-RADS 2, 21 mm ± 18 mm for LI-RADS 3, 13 mm ± 6 mm for LI-RADS 4 and 29 mm ± 13 mm for LI-RADS 5 (*p* < 0.001). Post hoc analysis showed a statistically significant difference in lesion size between LI-RADS 2 and LI-RADS 5 lesions.

### Diagnostic accuracy

The sensitivity, specificity, PPV and NPV for each reader per LI-RADS category are included in Table 1. All readers showed excellent specificities (88% – 100%) and NPVs (85% – 100%) across the LI-RADS categories. Sensitivities were variable, ranging from 67% to 83% for LI-RADS 1, 29% to 43% for LI-RADS 2, 100% for LI-RADS 3, 70% to 80% for LI-RADS 4 and 80% to 84% for LI-RADS 5. Readers misclassified 4/10 LI-RADS 4 cases as either LI-RADS 3 or LI-RADS 5 observations. Readers misclassified 6/25 LI-RADS 5 observations, with one observation allocated as LI-RADS 2 and the other 5 observations allocated as LI-RADS 4.

**TABLE 1:** The sensitivity, specificity, positive predictive value and negative predictive value per Liver Imaging Reporting and Data System category for each reader.

Variable	LI-RADS 1		LI-RADS 2		LI-RADS 3		LI-RADS 4		LI-RADS 5	
	%	95% CI	%	95% CI	%	95% CI	%	95% CI	%	95% CI
**Sensitivity**										
R1	83.3	35.9% – 99.6%	28.6	3.7% – 71.0%	100.0	15.8% – 100%	80.0	44.4% – 97.5%	80.0	44.4% – 97.5%
R2	83.3	35.9% – 99.6%	28.6	3.7% – 71.0%	100.0	15.8% – 100%	70.0	34.8% – 93.3%	84.0	63.9% – 95.5%
R3	66.7	22.3% – 95.7%	42.9	9.9% – 81.6%	100.0	15.8% – 100%	70.0	34.8% – 93.3%	84.0	63.9% – 95.5%
**Specificity**										
R1	97.7	88.0% – 99.9%	97.7	87.7% – 99.9%	89.6	77.3% – 96.5%	95.0	83.1% – 99.4%	95.0	83.1% – 99.4%
R2	97.7	88.0% – 99.9%	97.7	87.7% – 99.9%	87.5	74.8% – 95.3%	92.5	79.6% – 98.4%	92.0	74.0% – 99.0%
R3	100	92.0% – 100%	93.0	80.9% – 98.5%	91.7	80.0% – 97.7%	90.0	76.3% – 97.2%	92.0	74.0% – 99.0%
**PPV**										
R1	83.3	41.1% – 97.3%	66.7	17.2% – 95.1%	28.6	14.9% – 47.8%	80.0	50.0% – 94.1%	80.0	50.0% – 94.1%
R2	83.3	41.1% – 97.3%	66.7	17.2% – 95.1%	25.0	13.6% – 41.3%	70.0	42.2% – 88.2%	91.3	73.3% – 97.6%
R3	100		50.0	20.0% – 80.0%	33.3	16.4% – 56.1%	63.6	38.8% – 82.8%	91.3	73.3% – 97.6%
**NPV**										
R1	97.7	87.8% – 99.6%	89.4	84.0% – 93.1%	100.0	-	95.0	84.6% – 98.5%	95.0	84.6% – 98.5%
R2	97.7	87.8% – 99.6%	89.4	84.0% – 93.1%	100.0	-	92.5	82.7% – 97.0%	85.2	69.9% – 93.4%
R3	95.7	87.7% – 98.6%	90.9	84.0% – 95.0%	100.0	-	92.3	82.2% – 96.9%	85.2	69.9% – 93.4%

LIRADS, Liver Imaging Reporting and Data System; R1, reader 1; R2, reader 2; R3, reader 3; PPV, positive predictive value; NPV, negative predictive value; 95% CI, 95% confidence interval.

**FIGURE 1:**
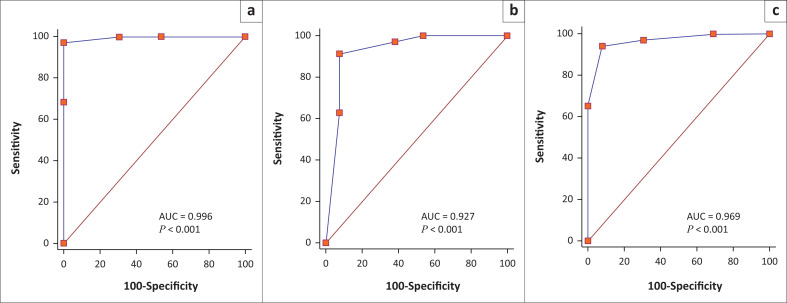
The receiver operating characteristic curve (ROC) for each reader for differentiating benign from malignant liver lesions. (a) R1; (b) R2; (c) R3.

The diagnostic accuracy of the readers as evaluated using ROC analysis is included in Figure 1. Based on reference standard, when accuracy for ‘benign’ (LI-RADS 1 and LI-RADS 2) versus ‘malignant’ (LI-RADS 4 and LI-RADS 5) is calculated, reader AUC values are as follows:

- R1, AUC of 0.99 (0.92 to 1.00), *p* < 0.001
- R2, AUC of 0.93 (0.81 to 0.98), *p* < 0.001
- R3, AUC of 0.97 (0.87 to 1.00), *p* < 0.001

Pairwise comparison of the ROC curves between R1 and R2 (*p* = 0.19), R1 and R3 (*p* = 0.18) and R2 and R3 (*p* = 0.27) showed no significant differences between individual reader performances.

### Inter-reader agreement

The overall inter-reader agreement for the three readers as a group was ‘good’ (κ = 0.76; 95% confidence interval [CI]: 0.67 to 0.85; *p* < 0.001). Kappa values for agreement on individual LI-RADS categories were ‘good’ or ‘very good’, as follows:

- LI-RADS 1, κ = 0.86 (0.70 to 1.00), *p* < 0.001
- LI-RADS 2, κ = 0.64 (0.48 to 0.80), *p* < 0.001
- LI-RADS 3, κ = 0.83 (0.67 to 0.99), *p* < 0.001
- LI-RADS 4, κ = 0.68 (0.5 to 0.84), *p* < 0.001
- LI-RADS 5, κ = 0.79 (0.63 to 0.95), *p* < 0.001

Pairwise inter-reader agreement, as evaluated using weighted kappa, was ‘very good’, as follows:

- R1 and R2, κ = 0.90 (0.79 to 1.00), *p* < 0.001
- R1 and R3, κ = 0.91 (0.81 to 1.00), *p* < 0.001
- R2 and R3, κ = 0.96 (0.93 to 0.99), *p* < 0.001

## Discussion

This study demonstrates excellent specificities (87% – 100%) and NPVs (85% – 100%) across the LI-RADS categories, coupled with excellent AUCs (0.93–0.99) indicating that accurate differentiation between benign and malignant liver lesions by individual readers is possible with LI-RADS version 2018. Despite the excellent diagnostic specificities, the sensitivities across the LI-RADS categories were more variable (28% – 100%). At least in part, this reflects the complexities in detecting and characterising lesions in the context of the cirrhotic liver. Lesion evaluation may prove to be challenging in circumstances including: (1) detection of small HCCs in a cirrhotic liver with innumerable regenerating nodules; (2) detection of small HCCs in a cirrhotic liver with multiple arterial enhancing perfusion anomalies or with transient hepatic intensity differences (THIDs); (3) regression and fibrosis occurring in benign lesions in the cirrhotic liver resulting in an atypical imaging appearance (e.g. hyalinised haemangiomas); and (4) development of pseudo-lesions that can confound interpretation (THIDs, confluent fibrosis, segmental or lobar dysmorphism). The variable sensitivity is mitigated by the fact that cirrhotic patients typically undergo regular follow up imaging every 3–6 months, thereby increasing the probability of lesion detection at a future examination.

In this study, the overall inter-reader agreement was ‘good’ (κ = 0.76), while pairwise inter-reader agreement was ‘very good’ (κ = 0.90, 0.91, 0.96). As with prior studies, we also found comparatively lower reproducibility in categorising LI-RADS 2 (κ = 0.64) and LI-RADS 4 (κ = 0.68) lesions and higher reproducibility for the LI-RADS 1 (κ =0.86), LI-RADS 3 (κ = 0.83) and LI-RADS 5 (κ = 0.79) categories.^6,9,10,12^ This may reflect greater reader confidence and agreement for assigning categories that denote either ‘definitively’ benign (LI-RADS 1) or ‘definitely’ malignant (LI-RADS 5) lesions versus categories that denote either ‘probably’ benign (LI-RADS 2) or ‘probably’ malignant (LI-RADS 4) lesions. Interestingly, our study showed very good agreement on categorising LI-RADS 3 lesions (κ = 0.83) using the updated LI-RADS version 2018 lexicon, although this may be due to the small number of LI-RADS 3 cases in our study (*n* = 2, 4%).

Several prior studies have also assessed reader agreement, mostly utilising earlier LI-RADS versions.^12,13,14,15,16,17,18^ A study by Davenport et al. found poor reproducibility (κ = 0.35) of the LI-RADS 2014 lexicon when they compared studies investigating other non-LI-RADS reporting systems. They found the highest level of agreement in LI-RADS 1 and LI-RADS 5 categories (κ = 0.54 and κ = 0.62, respectively). There was low reproducibility for LI-RADS 2, LI-RADS 3 and LI-RADS 4 categories, much lower than our study (κ = 0.11, 0.26, and 0.28, respectively).^16^ A more recent study comparing LI-RADS versions 2017 and 2018 also found that the updated LI-RADS 5 criteria of LI-RADS 2018 yielded significantly better sensitivity (81%) than LI-RADS 2017 (68%) for non-invasive diagnosis of HCC.^15^ A recent prospective cohort study by Razek et al. found excellent inter-observer agreement for LI-RADS 1, LI-RADS 2 and LI-RADS 5 using the 2018 LI-RADS lexicon.^12^ However, in their study, agreement was poor in LR-3 and LR-4. By contrast, two studies have previously observed good reproducibility for all LI-RADS categories by MRI (κ = 0.609 and κ = 0.926, respectively) using the LI-RADS version 2014 lexicon.^13,14^ The reproducibility differences in these previous studies may be related to a combination of reader factors (i.e. modality and readers’ liver imaging expertise, prior familiarity with the LI-RADS lexicon, number of years of post residency practice) and study factors (i.e. complexity of lesions, number of readers, whether readers were based at a single institution or multicenter international reader pool, including community practice, academic and mixed practice environments).

This study is subject to several limitations. Firstly, our selected reference standard was expert consensus without histological correlation. Validation of the LI-RADS risk stratification and management system has been shown in prior studies.^6,9,10,19^ In a recent systematic review and meta-analysis, Van der Pol et al. showed the likelihood of HCC in LI-RADS 2, LI-RADS 3, LI-RADS 4 and LI-RADS 5 categories was 13% (CI: 18–22), 38% (CI: 31–45), 74% (CI: 67–80) and 94% (CI: 92–96), respectively.^10^ A prospective study by Pinero et al. comparing LI-RADS categories with histopathology findings in liver transplant explants found a probability of HCC for LI-RADS 3, LI-RADS 4 and LI-RADS 5 of 50% (CI: 18–90), 89% (CI: 59–98) and 77% (CI: 64–87), respectively.^9^ Furthermore, a consensus reference standard has been used in studies evaluating previous LI-RADS versions.^15,19^ However, some lesions labelled as ‘benign’ in the LI-RADS 2 category may represent malignancy histologically.^6,10^ The American College of Radiology guidelines currently recommend serial surveillance for these lesions.^7^ Secondly, our study was limited to a testing bank of 50 cases spread across LI-RADS categories. It is possible that small but important differences may exist but were not identified by relatively large CIs in some LI-RADS categories. Thirdly, for practical testing purposes, we excluded some categories of observations including LR-TIV and LR-M, which may limit applicability in a prospective clinical environment. Fourthly, we used a non-consecutive selection of cases over our study period. Although this approach was chosen in an effort to acquire a variable set of LI-RADS observation types and imaging difficulty, it is possible that this approach can predispose to selection bias. Finally, our readers were fellowship trained body imaging radiologists with at least five years of clinical experience, which may limit generalisability. Despite these limitations, the authors believe that the rigorous study design and subsequent results of this study supports the applicability of LI-RADS version 2018 in appropriate cases and with the appropriate use of ACR management guidelines.

## Conclusion

This study demonstrates excellent specificity, NPV and AUC values of the LI-RADS version 2018 for risk stratification of focal liver observations, validating the use of version 2018 for differentiating benign from malignant lesions. Although inter-reader agreement was ‘good’, there remains ongoing reduced inter-reader agreement amongst intermediate LI-RADS categories, highlighting the need for the risk stratification tool to be used in conjunction with a conservative management recommendation system for high-risk patients.
